# Deprescribing opportunities for patients with advanced non-small cell lung cancer: an observational study of potentially inappropriate medications and stakeholders’ perspectives

**DOI:** 10.1007/s11096-026-02158-4

**Published:** 2026-05-13

**Authors:** Fenna de Vries, Eva Verbeek, Arthur Smit, Michel van den Heuvel, Rob ter Heine, Eric Franssen

**Affiliations:** 1 Department of Pharmacy, OLVG, Amsterdam, the Netherlands; 2 Department of Pharmacy, Pharmacology & Toxicology, Research Institute for Medical Innovation, Radboudumc, Nijmegen, the Netherlands; 3 Department of Cardiology and Cardiothoracic Surgery, OLVG, Amsterdam, the Netherlands; 4 Department of Pulmonology, OLVG, Amsterdam, the Netherlands; 5 Department of Pulmonology, University Medical Center Utrecht, Utrecht, the Netherlands; 6 Department of Respiratory Medicine, Research Institute for Medical Innovation, Radboudumc, Nijmegen, the Netherlands

**Keywords:** Deprescribing, Medication optimization, Non-small cell lung cancer, Polypharmacy, Potentially inappropriate medication

## Abstract

**Introduction:**

Polypharmacy (concurrent use of ≥ 5 medications) is common in patients with non-small cell lung cancer and increases the risk of potentially inappropriate medication use, particularly in those with advanced disease. Deprescribing may reduce medication-related harm; however, its implementation in oncology remains limited and is insufficiently informed by stakeholder perspectives.

**Aim:**

This study aimed to determine the prevalence of potentially inappropriate medications in patients with advanced non-small cell lung cancer and explore the attitudes and beliefs of patients, caregivers, and healthcare professionals regarding deprescribing to inform feasible implementation strategies for routine clinical practice.

**Method:**

A retrospective analysis was conducted in a cohort of 817 patients with stage IV non-small cell lung cancer to identify potentially inappropriate medications using the OncoSTRIP checklist. Perspectives on deprescribing were collected using validated questionnaires: the revised Patients’ Attitudes Toward Deprescribing (rPATD) for patients and caregivers and the Comprehensive Healthcare Providers’ Opinions, Preferences, and Attitudes Toward Deprescribing (CHOPPED) for medical clinicians and pharmacists, which were completed by 37 patients, 15 caregivers, 24 medical clinicians (physicians and specialist nurses), and 24 pharmacists.

**Results:**

Potentially inappropriate medications were algorithm-flagged in 793 of the 817 (97.1%) patients. Among these, 1,686 (40.8%) were confirmed by clinical evidence, while 2,448 (59.2%) were unconfirmed (defined as medications flagged by the checklist but lacking sufficient electronic health record data, such as laboratory values or specific indications, to definitively justify their use). The primary classifications were the risk of adverse drug events (47.3%) and unnecessary therapy (30.0%). Most patients (33, 89.2%) and caregivers (11, 73.3%) were willing to discontinue their medications if advised by a physician. Perceived barriers influenced medical clinicians’ and pharmacists’ willingness: medical clinicians were most affected by collaboration- and competence-related concerns, whereas pharmacists were constrained by competence- and patient-related factors.

**Conclusion:**

The high algorithm-flagged prevalence of potentially inappropriate medications with 40.8% confirmed by clinical evidence, in patients with advanced non-small cell lung cancer underscores the urgent need for deprescribing. Successful implementation requires a patient-centered approach, active involvement of patients and caregivers, interprofessional collaboration, and targeted strategies to address systemic and professional barriers to ensure safe and sustainable medication optimization.

**Supplementary Information:**

The online version contains supplementary material available at https://doi.org/10.1007/s11096-026-02158-4.

## Impact statements

- Potentially inappropriate medications (PIMs) are highly prevalent in advanced non-small cell lung cancer with specific drug classes such as dyslipidemics antimicrobials and proton pump inhibitors showing high rates of clinical support for deprescribing.
- Distinguishing between algorithm-flagged and clinically confirmed PIMs is essential for accurate prevalence reporting and for prioritizing medications for clinical review.
- Patient and caregiver engagement is critical with shared decision-making supporting safe deprescribing of PIMs.
- Addressing clinician and pharmacist barriers enables patient-centered sustainable strategies for medication optimization and the management of PIMs in oncology workflows

## Introduction

At the time of diagnosis, more than half of patients with non-small cell lung cancer (NSCLC) present with stage IV disease [1]. These patients are typically between 65 and 70 years of age and often have multiple chronic conditions, such as hypertension, hypercholesterolemia, and metabolic syndrome [2–4]. Individuals with lung cancer are also twice as likely to have comorbidities as those without cancer, and more than 65% of patients with advanced NSCLC have at least one comorbidity [5–11]. The management of these conditions requires pharmacological treatment, resulting in polypharmacy (concurrent use of five or more drugs) in nearly half of this population [11, 12].

While managing chronic conditions with medication can help prevent complications, it also increases the risk of prescribing potentially inappropriate medications (PIMs), which may cause harm [13–16]. PIMs are generally linked to adverse drug events, increased healthcare use, reduced functional capacity, and a decline in overall quality of life [17–20]. This impact is particularly profound in patients with advanced NSCLC, in whom PIMs increase the risk of drug–drug interactions, adverse events, and treatment-related toxicities. They also drive higher rates of unexpected hospitalizations and longer hospital stays, ultimately compromising overall survival [21–25]. This issue may be further complicated by the transition of care from general practitioners to specialist providers: specialists often hesitate to deprescribe medications, viewing it as the general practitioner’s responsibility [26]. This creates a gap in care in which opportunities to optimize medication may be missed after a terminal diagnosis. In addition, the limited median overall survival in real-world populations with metastatic NSCLC, ranging from approximately 3 months with best supportive care to 18 months with systemic therapy, underscores the need to evaluate pharmacological treatment at diagnosis [4, 27–29].

This complex interplay of advanced age, comorbidities, limited prognosis, and risk of inappropriate medication use highlights the need for a proactive treatment approach in this population. In this context, deprescribing has emerged as a promising patient-centered strategy. Deprescribing refers to the planned and supervised process of tapering or discontinuing medications that are no longer beneficial or may pose harm.

However, limited evidence on the prevalence of PIMs in this population makes it challenging to identify PIMs and integrate deprescribing into routine oncology practice. Moreover, sustainable deprescribing requires more than clinical insight alone; it depends on the active participation of patients, caregivers, and healthcare professionals. Currently, little is known about the beliefs and decision-making dynamics influencing this process, including psychological resistance to modifying established treatment regimens and the emotional complexities associated with terminal illness.

### Aim

Therefore, This study aimed to determine the prevalence of PIMs in patients with advanced NSCLC and to explore the attitudes and beliefs of key stakeholders, patients, caregivers, physicians, (specialist) nurses, and pharmacists regarding deprescribing, to inform feasible implementation strategies for routine clinical practice.

## Method

### Identification of PIMS

#### Study population and data collection

A retrospective study was conducted at OLVG Hospital (Amsterdam, the Netherlands) to identify PIMs in patients with advanced NSCLC. Patients using at least one medication at the time of advanced NSCLC diagnosis were identified using institutional data from the Dutch Lung Cancer Audit–Lung Carcinoma (DLCA-L), a national quality registry coordinated by the Dutch Institute for Clinical Auditing. The DLCA-L dataset was filtered to include patients newly diagnosed with stage IV NSCLC between 2017 and 2023.

#### Deprescribing tool

PIMs were identified using the “OncoSTRIP List of Drugs Suitable for Deprescribing in Older Cancer Patients” (hereafter, the OncoSTRIP checklist), which provides evidence-based justifications for PIMs [30]. This checklist was deemed most appropriate for our study population and research question. To apply the algorithm systematically, we assumed a median life expectancy of less than two years for this cohort. Patient mortality data were therefore tracked and reported to provide clinical context and to validate the appropriateness of applying OncoSTRIP criteria to this advanced NSCLC population.

To assess the presence of PIMs at the time of advanced NSCLC diagnosis, all medications (including over-the-counter (OTC) medications, if documented) from the electronic health record (EHR; Epic Systems Corporation, Verona, WI, USA) were analyzed alongside laboratory and clinical data collected within 6 months of diagnosis. Detailed information, including product type, dosage, and duration of each medication, was collected and verified for accuracy. A pharmacological recommendation was formulated for each item on the OncoSTRIP checklist using a computerized algorithm without additional pharmacist review. This approach aimed to systematically identify PIMs based on criteria such as contraindications and ineffective treatments. Medications were classified as confirmed PIMs when clear evidence of inappropriateness was available, and as unconfirmed PIMs when clinical context was lacking. For example, thiazide diuretics were flagged as inappropriate in the presence of hypokalemia but considered appropriate with normal potassium levels. When a single medication appeared in multiple checklist items, it was recorded as a PIM if flagged in any of them.

#### Data analysis

Patient characteristics, including age, sex, year of diagnosis, and survival status, were extracted from the EHR. The number of medications on the OncoSTRIP checklist, together with the presence, frequency, and classification of identified PIMs by drug class, was recorded. PIMs were categorized as confirmed or unconfirmed, and each case was analyzed for the motivation behind the flag or the missing clinical context. Medications initiated around the time of diagnosis were classified as chronic (initiated before the first outpatient visit) or acute (initiated between the first visit and diagnosis). Patient characteristics (age, medications) are reported as median [interquartile range, IQR] to reflect individual-level distributions. In contrast, overall survival is reported as median [95% confidence interval, CI], with the confidence interval reflecting the precision of the Kaplan–Meier estimate. All statistical analyses were conducted using R Statistical Software (v4.4.1) in RStudio (v2024.4.2.764) [31, 32].

### Perspective regarding Deprescribing of patients, caregivers, and healthcare professionals

#### Study population and data collection

The revised Patients’ Attitudes Towards Deprescribing (rPATD) questionnaire was administered to patients with stage IV NSCLC and their caregivers, while the Comprehensive Healthcare Providers’ Opinions, Preferences, and Attitudes Towards Deprescribing (CHOPPED) questionnaire was distributed to healthcare professionals treating these patients [33, 34]. Both questionnaires are validated and use a 5-point Likert scale (ranging from “Fully disagree” to “Fully agree”). The items “I spend a lot of money on my medicines” (patients) and “My care recipient’s medicines are quite expensive” (caregivers) were removed from the rPATD questionnaire, as they did not apply to the Dutch healthcare context.

Participants were recruited between August 2024 and March 2025. Within the pulmonology departments of OLVG Hospital (Amsterdam, the Netherlands) and Radboudumc Hospital (Nijmegen, the Netherlands), (specialist) nurses approached eligible patients and caregivers to discuss participation. Potential participants could either scan a provided QR code for direct access to the rPATD questionnaire or leave their email address with the nursing staff, after which the coordinating researcher would send an invitation email containing a link to the questionnaire. Healthcare professionals, including pharmacists and medical clinicians (a term used hereafter to refer collectively to physicians and specialist nurses), from the pulmonology and pharmacy departments of both hospitals, and from the researchers’ professional network, were recruited via email with a link to the CHOPPED questionnaire.

Eligible participants were adults able to complete an online questionnaire in Dutch independently. Patients were required to have a recent diagnosis of advanced NSCLC and to use at least one chronically prescribed medication. Caregivers were defined as any non-professional (e.g., family member, friend, or neighbor) providing regular physical, logistical, or decisional support to a patient with advanced NSCLC, regardless of cohabitation status. Healthcare professionals were required to be directly involved in the treatment of patients with advanced NSCLC. Because progress could not be saved during completion, questionnaires had to be finished in a single session, and only fully completed questionnaires were included in the analysis.

#### Data analysis

Participant characteristics were described using descriptive statistics. Following the scoring methodologies established for the rPATD and CHOPPED questionnaires, factor scores were calculated by averaging individual item scores within each domain (e.g., burden, appropriateness, and concerns) to obtain a composite measure of the underlying construct. Consistent with prior rPATD and CHOPPED analyses, chi-squared tests were used for frequency comparisons, and the Mann–Whitney U test was used to evaluate differences in factor scores between groups. Gamma rank correlation was used to assess associations between factor scores and the primary outcome of “willingness to deprescribe”, with *p*-values < 0.05 considered significant. This non-parametric measure was preferred because the Likert-scale data are ordinal and the analysis needed to account for the high number of tied ranks typical of such stakeholder surveys [35, 36].

Given the exploratory nature of the stakeholder survey, no formal sample size calculation was performed, and inferential analyses are hypothesis-generating. Data quality was assessed using Cronbach’s alpha, with values ≥ 0.70 indicating acceptable reliability [37]. Robustness was determined by bootstrap mean-stability analysis, with confidence intervals < 10% of the mean score considered robust [38]. Rater agreement was measured using the intraclass correlation coefficient, with values < 0.5 indicating poor reliability and > 0.90 indicating excellent reliability [39]. All statistical analyses were performed using R Statistical Software (v4.4.1) within RStudio (v2024.4.2.764) [31, 32].

### Ethics approval

The study was exempted from the Medical Research Involving Human Subjects Act (WMO) by the Medical Ethics Committees United (local registry number WO24.072); formal ethics approval and written informed consent were therefore neither required nor obtained. For the prospective component on perspectives regarding deprescribing, an informed consent form was included in the questionnaire, and participants were required to provide consent to participate and to publish the results.

## Results

### Identification of PIMS

#### Study population

A total of 920 patients were diagnosed between 2017 and 2023, of whom 103 had no drug use at diagnosis, leaving 817 patients in the final study population. The median age was 68 years (IQR 61–75), and 57.8% of patients were male (Table 1). Laboratory and clinical parameters were mainly within normal limits (Supplementary Table 1). Patients used a median of six chronic medications (IQR 3–9) listed in the OncoSTRIP checklist, with 514 (62.9%) meeting the polypharmacy criterion. At the data cut-off of 1 May 2025, 113 patients (13.8%) were alive. Median overall survival was 148 days (95% CI 129–183), with notable differences between patients receiving best supportive care (OS 43 days, 95% CI 37–51) and those receiving active treatment (OS 331 days, 95% CI 277–416).

**Table 1:** Characteristics of the Study Population (N = 817)

	Categorical variables *N* (%)	Continuous variables median [IQR]
Baseline characteristics		
*N*	817	
Age (years)		68 [61–75]
Sex		
Female	345 (42.2)	
Male	472 (57.8)	
Year of diagnosis		
2017	94 (11.5)	
2018	100 (12.2)	
2019	140 (17.1)	
2020	105 (12.9)	
2021	133 (16.3)	
2022	124 (15.2)	
2023	121 (14.8)	
Time between first visit and diagnosis (days)		8 [2–16]
Number of medications included in the OncoSTRIP checklist per patient		6 [3–9]
Polypharmacy	514 (62.9)	
Treatment		
Anti-tumor (palliative)	424 (51.9)	
Best supportive care	274 (33.5)	
Unknown	119 (14.6)	
Characteristics at cut-off^#^		
Survival status		
Alive	113 (13.8)	
Dead	704 (86.2)	
Overall survival, days*		
Overall		148 [129–183]
Anti-tumor (palliative)		331 [277—416]
Best supportive care		43 [37—51]
Unknown		244 [181—385]

^*^Data are presented as median [95% CI]. ^#^Data cut-off established as May 1, 2025

A total of 5,276 prescriptions were extracted from the EHR, with most patients using prescriptions for gastrointestinal disorders (63.3%), pain (50.9%), antithrombotic therapy (50.1%), or cardiovascular disorders (47.7%).

#### (Potentially) inappropriate medications

**Fig. 1:**
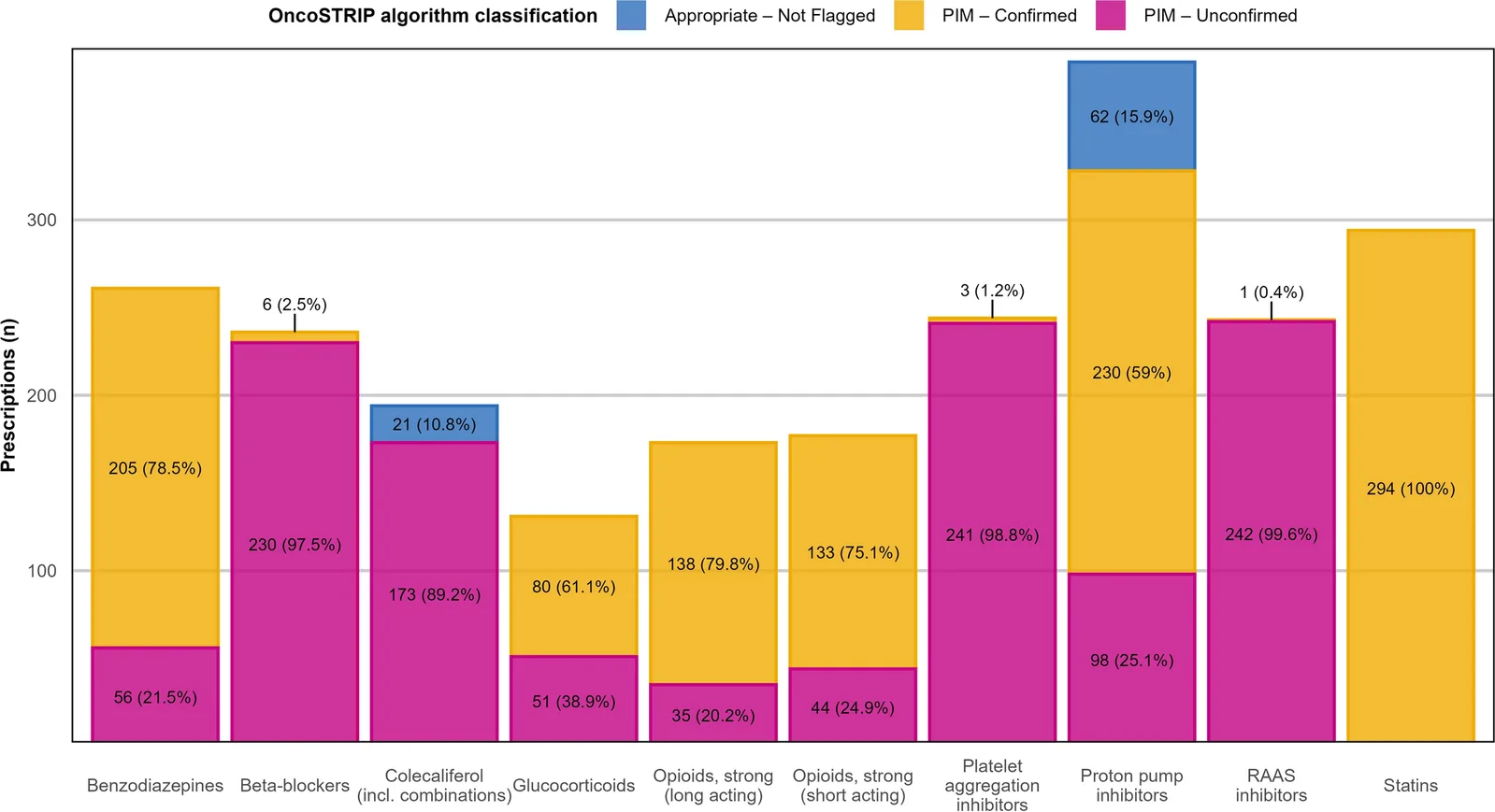
Prescriptions Most Frequently Flagged as PIM by the OncoSTRIP Algorithm. Numbers and proportions of prescriptions within each drug group flagged by the computerized oncoSTRIP tool as either “Appropriate”, “Confirmed” PIM (supported by clinical data) or “Unconfirmed” PIM (lacking sufficient clinical for appropriateness) *Excluding the weak opioids tramadol, codeine, and their combination products

In total, 4,134 PIMs were flagged by the computerized OncoSTRIP algorithm in 793 of 817 patients (97.1%). An overview of all prescription data and the flagged PIMs per patient is presented in Supplementary Table 2. Of the flagged PIMs, 1,686 (40.8%) were classified as potentially inappropriate based on available clinical information, while 2,448 (59.2%) lacked sufficient clinical context to confirm their appropriateness. The ten drug groups with the highest number of identified PIMs are shown in Fig. 1 (a comprehensive overview is provided in Supplementary Table 3).

Among confirmed PIMs, the most common adverse drug event risk was observed with antimicrobials (100%), allergy medications (84%), and analgesics (74%). Unnecessary drug therapy was the sole reason for anti-dyslipidemia drugs (100%) and was often identified in antidiabetics (45%) (Supplementary Fig. 1a). For unconfirmed PIMs, the main gaps concerned treatment indication and efficacy, especially in endocrine (77%), vitamins/minerals/OTC (72%), and antidiabetic (66%) medications. Additional data gaps included concurrent symptoms/comorbidities in respiratory (53%) and nervous system (43%) medications, and laboratory/physiological measurements in anti-inflammatories (38%) and analgesics (32%) (Supplementary Fig. 1b).

Initiation patterns varied substantially across drug groups and screening categories (Supplementary Fig. 2). Heparin and antiemetics were predominantly initiated recently, regardless of PIM status. In contrast, proton pump inhibitors and platelet aggregation inhibitors flagged as PIMs were almost exclusively initiated for chronic use. Glucocorticoids and opioids showed a more balanced distribution between chronic and recent use. For several medication classes, including direct oral anticoagulants (DOACs) and NSAIDs/coxibs, confirmed PIM status was more frequently linked to recent initiation than unconfirmed PIM status.

### Perspective regarding Deprescribing of patients, caregivers, and healthcare professionals

#### Participant characteristics

The patient questionnaire was accessed 73 times through either the QR code or hyperlink. Of these, 37 resulted in completed questionnaires, 5 were initiated but not completed, and 31 were not commenced after the link was opened. Similarly, the caregiver questionnaire was accessed 28 times, yielding 15 completed responses, 4 incomplete, and 9 not initiated. Patients who completed the questionnaire had a median age of 71 years (IQR 64–76), and caregivers had a median age of 67 years (IQR 62–71) (Table 2). Most patients were male (59.5%), whereas caregivers were predominantly female (60.0%). Polypharmacy was prevalent, with 43.2% of patients using more than five medications, and 86.5% of patients managing their medications independently. The most common comorbidities were cardiovascular disease (51.4%) and musculoskeletal disorders (35.1%).

**Table 2:** Characteristics of the Participants of the rPATD Questionnaire (*N* = 52)

Characteristics	Patients	Caregivers
	*N* (%)*	*N* (%)*
*N*	37	15
Age, years (median, IQR)	71 [64–76]	67 [62–71]
Sex		
Female	15 (40.5)	9 (60.0)
Male	22 (59.5)	6 (40.0)
Number of regular medications		
1–2	8 (21.6)	
3–5	13 (35.2)	
6–10	9 (24.3)	
11–15	5 (13.5)	
16–20	1 (2.7)	
> 20	1 (2.7)	
Comorbidities^#^		
None	5 (13.5)	
High or low blood pressure, angina, heart attack or heart failure	19 (51.4)	
Stroke (blood clot or bleeding in the brain)	6 (16.2)	
Inflammatory bowel disease, reflux disease or stomach ulcers	2 (5.4)	
Diabetes	3 (8.1)	
Kidney disease	2 (5.4)	
Chronic obstructive lung disease, asthma or chronic bronchitis	9 (24.3)	
Arthritis or chronic back pain	13 (35.1)	
Osteoporosis	4 (10.8)	
Memory problems	1 (2.7)	
Relationship of care recipient		
Spouse/de facto		11 (73.4)
Mother/father		2 (13.3)
Mother/father-in-law		
Sibling		2 (13.3)
Other relative		
Other non-relative		
Residence of care recipient		
At home by themselves	15 (40.5)	
At home with family/friends	22 (59.5)	
At home with carer		
Medication management		
Self-manage	32 (86.5)	
Self-manage with assistance from family/friend	4 (10.8)	
Family/friend	1 (2.7)	
Paid care		
Education level		
Secondary or vocational	29 (78.4)	11 (73.3)
Higher education	8 (21.6)	4 (26.7)

^*^Data are presented as frequencies and percentages unless otherwise specified

^#^Multiple answers possible

Healthcare professionals used a single generic hyperlink, accessed it 107 times, and completed 48 questionnaires (24 from medical clinicians and 24 from pharmacists). In addition, 12 sessions were started but not completed (four by each group), and 47 sessions ended before the questionnaire began. Participant characteristics are detailed in Table 3.

**Table 3:** Characteristics of the Participants of the CHOPPED Questionnaire (*N* = 48)

Characteristics	Medical clinicians	Pharmacists
	*N* (%)*	*N* (%)*
*N*	24	24
Age, years (median, IQR)	49 [44—53]	38 [33–46]
Sex		
Female	14 (58.3)	17 (70.8)
Male	10 (41.7)	7 (29.2)
Medical position		
Community pharmacist		6 (25.0)
Hospital pharmacist		11 (45.8)
No specialization		
In specialist training	2 (8.3)	7 (29.2)
General practitioner	1 (4.2)	
Pulmonologist	19 (79.2)	
Specialized nurse	2 (8.3)	
Workplace^#^		
Academic hospital	13 (52)	11 (40.7)
Community pharmacy		
Top clinical hospital	8 (32)	15 (55.6)
General hospital	3 (12)	1 (3.7)
Primary care/general practice	1 (4)	
Years of experience (median, IQR)	20 (14—25)	12 (7—20)
Highest educational attainment		
Medical education (University of applied sciences)	1 (4.2)	
Medical education (University)	2 (8.3)	6 (25.0)
Medical specialization (after University of applied sciences)	1 (4.2)	
Medical specialization (after University)	9 (37.5)	10 (41.7)
PhD program	11 (45.8)	5 (20.8)
Postdoctoral research		3 (12.5)

^*^Data are presented as frequencies and percentages unless otherwise specified

^#^Multiple answers possible

#### Willingness to Deprescribe

Overall, patients showed a strong willingness to stop medications if their clinician suggested it (89.2% agreement, 5.4% uncertain, 5.4% disagreement), whereas caregivers were less certain (73.3% agreement, 20.0% uncertain, 6.7% disagreement). All medical clinicians (100.0%) were willing to deprescribe. Among pharmacists, 83.3% agreed, and 16.7% reported uncertainty.

**Fig. 2:**
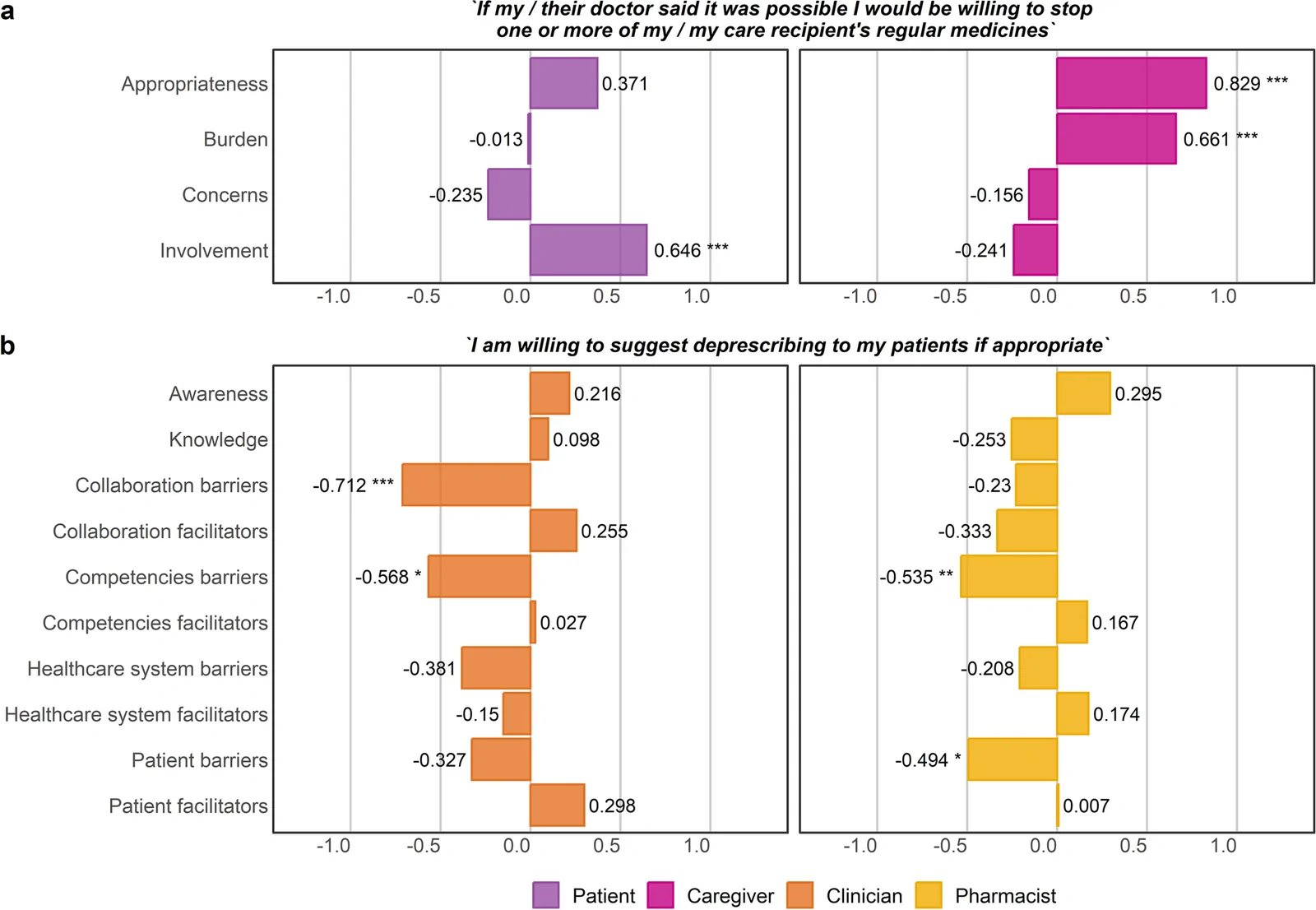
Factors Influencing Willingness to Deprescribe. Data are presented as Gamma (γ) rank correlation coefficients. Factors influencing willingness to deprescribe among patients and caregivers (**a**), and factors influencing willingness to suggest deprescribing among medical clinicians and pharmacists (**b**). ***p < 0.001, **p < 0.01, *p < 0.05

Gamma rank analysis showed that patients’ involvement in medication management correlated with willingness to deprescribe (γ = 0.646, *p* < 0.001) (Fig. 2a). For caregivers, willingness was associated with higher appropriateness concerns (γ = 0.829, *p* < 0.001) and perceived medication burden (γ = 0.661, *p* < 0.001). Among medical clinicians, willingness to suggest deprescribing was negatively associated with perceived collaboration (γ =  − 0.712, *p* < 0.001) and competence barriers (γ =  − 0.568, *p* = 0.011) (Fig. 2b). Other barriers, patient-related (γ =  − 0.327, *p* = 0.217) and healthcare system–related (γ =  − 0.381, *p* = 0.099), were not significantly associated. For pharmacists, willingness to deprescribe decreased with competence-related (γ =  − 0.535, *p* = 0.003) and patient-related (γ =  − 0.494, *p* = 0.019) barriers.

#### Perceptions, barriers, and facilitators of medication use and deprescribing

Many patients (40%) and caregivers (27%) believed medications were unnecessary, while 17% of patients and 27% of caregivers considered them ineffective; 46% of patients and 66% of caregivers considered them harmful. Caregivers perceived a higher medication burden (40% vs. 24% of patients; U = 388.5, Z = 2.242, *p* = 0.025). Patients were more involved in medication management (92.0% vs. 66.0%; χ^2^ = 9.96, *p* = 0.041), confirmed by factor scores (U = 171.0, Z =  − 2.151, *p* = 0.030). Although 87% of both groups did not equate deprescribing with withdrawal of care, over half of caregivers (60.0%) and 43.2% of patients were hesitant to stop long-term medications.

Patients’ satisfaction with medications correlated positively with involvement (γ = 0.623, *p* < 0.001) and negatively with perceived burden (γ =  − 0.540, *p* = 0.001), lower perceived appropriateness (γ =  − 0.512, *p* = 0.002), and concerns (γ =  − 0.413, *p* = 0.014). For caregivers, lower perceived burden was associated with lower satisfaction (γ =  − 0.571, *p* = 0.008). Both medical clinicians and pharmacists showed strong awareness of the value of deprescribing: 100.0% of pharmacists and 80.0% of medical clinicians agreed that it is as important as prescribing. Most medical clinicians (83.3%) supported pharmacist involvement, 66.7% of pharmacists believed physicians would accept their recommendations, and 74.0% disagreed that such recommendations would damage relationships (χ^2^ = 19.64, *p* = 0.001). However, interprofessional tensions persisted: 58.3% of medical clinicians considered it inappropriate to suggest stopping medications prescribed by other physicians, and 33.3% considered it inappropriate for another physician to suggest stopping medications they had prescribed.

System-level barriers, such as fragmented care, were recognized more often by medical clinicians (63.0%) than by pharmacists (45.0% disagreement; χ^2^ = 12.20, *p* = 0.016). Financial support for deprescribing was endorsed by 60.0% of pharmacists and 40.0% of medical clinicians (χ^2^ = 12.53, *p* = 0.014). Both groups supported competency facilitators, including educational and clinical tools, and both identified long-term medication use as a challenge (medical clinicians, 55.0%; pharmacists, 53.0%). Pharmacists showed higher knowledge scores (U = 386.5, Z = 2.03, *p* = 0.041) and greater endorsement of system-level facilitators (U = 192.5, Z =  − 1.97, *p* = 0.048). Patient- and competency-related barriers reduced their willingness to suggest deprescribing (γ =  − 0.494, *p* = 0.019; γ =  − 0.535, *p* = 0.003). Collaboration- and competency-related barriers were most strongly associated with medical clinicians’ willingness (γ =  − 0.712, *p* < 0.001; γ =  − 0.568, *p* = 0.011).

Additional insights into the outcomes for each question, factor scores, and differences between respondents of the rPATD and CHOPPED questionnaires are provided in Supplementary Figs. 3.1, 3.2, 4.1a–c, and 4.2.

#### Data quality and reliability

The internal consistency was acceptable, with Cronbach’s alpha ranging from 0.81 to 0.85 across participant groups (patients: 0.83; caregivers: 0.84; clinicians: 0.81; pharmacists: 0.85). Bootstrap analysis revealed stable mean scores (3.02–3.34) and low standard errors (0.056–0.066). However, inter-rater reliability was poor, with intraclass correlation coefficients below 0.08 for all groups (patients: 0.064; caregivers: 0.020; clinicians: 0.049; pharmacists: 0.045), indicating high rating variability. Further details are in Supplementary Table 4.

## Discussion

### Identification of PIMS

This study evaluates PIMs and stakeholder views on deprescribing in patients with advanced NSCLC. Although the algorithm flagged 97.1% of patients, clinical evidence confirmed a PIM prevalence of 40.8%, which aligns with the 35–78% range previously reported [23, 24, 30, 40–44]. The remaining 59.2% of flags could not be verified due to insufficient clinical context, indicating that the algorithm functions as a high-sensitivity screening signal rather than definitive evidence of inappropriateness. Because our analysis relies entirely on an algorithm without external validation, it should be regarded as a screening approach that may overestimate PIM prevalence. Nevertheless, the findings reveal considerable room for improvement of polypharmacy in advanced lung cancer, while underscoring that outcomes depend on both the specific algorithm applied and the available clinical information. Identifying PIMs should therefore be viewed as an initial screening step rather than a conclusive clinical judgment. Deprescribing decisions must always incorporate patient-specific factors such as prognosis and individual care goals. For instance, while a proton pump inhibitor may be flagged as inappropriate without a documented indication, clinicians may reasonably continue its use when compelling reasons exist, such as an elevated risk of gastrointestinal bleeding.

The types of PIMs identified in this cohort support the clinical plausibility of the detected signals. Adverse drug events and unnecessary treatments were primary motivations for deprescribing, reflecting findings from existing oncology and geriatric studies. [14, 24, 30]. This pattern is particularly meaningful in advanced NSCLC, where limited life expectancy shifts the risk–benefit balance away from long-term preventive treatments. The clearest opportunities for deprescribing, therefore, emerged among chronic preventive therapies that were algorithm-flagged and clinically confirmed at high rates: dyslipidemia drugs (100% of flagged prescriptions confirmed), proton pump inhibitors without a documented indication (70.1%), and long-term benzodiazepines (78.5%).

Interpreting these signals, however, requires attention to both directions of potential misclassification. On one hand, some flagged medications, particularly short-term symptom-driven treatments initiated shortly before diagnosis, may remain clinically appropriate despite meeting formal PIM criteria, which could inflate the apparent burden. On the other hand, incomplete capture of over-the-counter medications and drugs outside the OncoSTRIP scope may have led to underestimation of true inappropriate use. To keep this evaluation as comprehensive as possible, we deliberately chose not to pre-filter EHR medications before applying OncoSTRIP, accepting a broader initial signal in exchange for a fuller picture of real-world medication use.

### Perspective regarding Deprescribing of patients, caregivers, and healthcare professionals

The questionnaire findings underscore the importance of patient-centered deprescribing. Although responses showed reliable group-level patterns, substantial individual variability remained, and small, uneven sample sizes mean associations should be regarded as exploratory rather than causal. The reported response rate reflects the number of times the questionnaire link was opened, so the actual response rate could be higher, though this does not affect interpretation.

Most patients (89.2%) were willing to stop medications if advised by their physician. In contrast, caregivers showed slightly lower willingness (73.3%), consistent with their greater perceived medication burden and stronger concerns about medication appropriateness. Caregiver willingness was positively associated with these concerns, whereas patient willingness was associated with greater involvement in medication management. This suggests caregiver willingness may reflect risk–benefit considerations, while patient willingness depends more on engagement in treatment decisions; consistent with previous research [36, 45–47].

Despite recognizing that some medications may be unnecessary or harmful, many respondents remained hesitant to discontinue long-term therapies (43.2% of patients; 60.0% of caregivers). Most, however, rejected the notion that deprescribing represents a withdrawal of care. These findings highlight the importance of shared decision-making, clear communication about treatment goals, and caregiver involvement when addressing medication burden in advanced cancer.

Healthcare professionals expressed strong conceptual support for deprescribing, with most agreeing that it is as important as prescribing. Distinct barriers, however, shaped their willingness to initiate discontinuation. For medical clinicians, reluctance was most strongly linked to collaboration- and competence-related concerns, the latter referring to perceived gaps in knowledge, skills, or confidence to justify deprescribing decisions, reflecting uncertainty about clinical appropriateness and hesitation to discontinue medications initiated by other prescribers. Similar findings have been reported previously, suggesting that additional training and support may increase confidence in medication review [26, 35, 48].

Pharmacists, in contrast, reported primarily system- and patient-related barriers, including limited time, reimbursement constraints, and restricted access to complete medical records, echoing earlier reports on structural barriers to pharmacist-led interventions [26]. Encouragingly, our findings point to growing acceptance of pharmacist involvement: most medical clinicians supported pharmacist participation, and most pharmacists expected their recommendations to be accepted by physicians, signaling an evolving culture of interprofessional collaboration.

From an implementation perspective, several opportunities emerge. Structured medication reviews could be embedded at key clinical transition points, particularly at diagnosis of advanced disease, when treatment goals are typically reassessed. Because barriers differ across drug classes, preventive therapies facing different hurdles than symptom-driven ones, strategies should be tailored to specific medication groups. EHR-based screening tools support identification through automated alerts for frequently inappropriate medications, and integrating medication reviews into multidisciplinary team discussions strengthens collaboration among oncologists, pharmacists, and primary care physicians, ensuring decisions align with prognosis and patient preferences.

However, the generalizability of these findings must be considered within the Dutch healthcare context. Pharmacists hold a structurally embedded role in medication review, with established pathways to general practitioners, which may facilitate deprescribing relative to systems with more limited pharmacist roles. Near-universal insurance coverage eliminates the financial burden from medication decisions, and a strong primary care infrastructure, with GP gatekeeping, shapes how prescribing responsibility is shared and how care transitions are managed. In systems with fragmented coverage or direct specialist access, the barriers and willingness patterns observed here may manifest differently. Nonetheless, the core challenges, high medication burden, variability in stakeholder attitudes, and the importance of interprofessional collaboration are likely applicable across diverse healthcare systems.

## Conclusion

This study demonstrates a high prevalence of algorithm-identified PIMs in patients with advanced NSCLC, highlighting the multifaceted challenges of deprescribing in real-world oncology practice. Computerized screening tools such as OncoSTRIP can surface medication optimization opportunities, but determining appropriateness requires careful consideration of individual patient context and shared decision-making. While patients and caregivers are generally receptive to optimization, healthcare professionals face significant organizational and collaboration-related barriers. These findings emphasize the importance of patient and caregiver engagement, interprofessional collaboration, and supportive system structures, including pharmacist-led medication reviews, integration of deprescribing tools into electronic prescribing systems, and improved communication among professionals, to enable effective and actionable deprescribing.

Future research should prioritize larger, multi-center longitudinal studies to validate these exploratory findings across diverse healthcare systems. Beyond stakeholder attitudes, focus must shift toward the practical implementation of automated screening tools within clinical workflows. Investigating the impact of pharmacist-led interventions on patient-reported outcomes and survival is essential to move from identifying potential signals to establishing a robust, evidence-based framework for actionable deprescribing in advanced cancer care.

## Supplementary Information

Below is the link to the electronic supplementary material.

Supplementary file1 (PDF 2739 KB)

## Data Availability

The datasets generated and/or analyzed during the current study are provided within the manuscript or supplementary information files.
