# Supplementary material for: Deprescribing opportunities for patients with advanced non-small cell lung cancer: an observational study of potentially inappropriate medications and stakeholders’ perspectives
Source: Int J Clin Pharm. 2026 May 13;48(5):1858–70. doi: 10.1007/s11096-026-02158-4 (PMC13569502; doi:10.1007/s11096-026-02158-4)
Supplement: Supplementary file 1 [file 11096_2026_2158_MOESM1_ESM.pdf]

Supplementary Material

Deprescribing opportunities for patients with advanced non-small cell lung cancer: An observational study of potentially inappropriate medications and stakeholders' perspectives

International Journal of Clinical Pharmacy

Fenna de Vries*, Eva Verbeek, Arthur Smit, Michel van den Heuvel, Rob ter Heine, Eric Franssen

* Department of Pharmacy, OLVG, Amsterdam, the Netherlands
Department of Pharmacy, Research Institute for Medical Innovation, Radboudumc, Nijmegen, the Netherlands
f.devries@olvg.nl / fenna.devries1@radboudumc.nl

Content

Supplementary Tables

- Supplementary Table 1 — Laboratory and Clinical Parameters
- Supplementary Table 2 — Prescription Data and Identified PIMs
- Supplementary Table 3 — Classification of Flagged PIMs
- Supplementary Table 4 — Data Quality and Reliability

Supplementary Figures

- Supplementary Fig. 1 — Clinical Rationale and Data Limitations for Identified PIMs
- Supplementary Fig. 2 — Temporal Initiation Patterns of Medications Flagged by the OncoSTRIP Algorithm.
- Supplementary Fig. 3.1 — Outcomes of the rPATD Questionnaire: Outcomes per Question
- Supplementary Fig. 3.2 — Outcomes of the rPATD Questionnaire: Outcomes per Factor
- Supplementary Fig. 4.1a — Outcomes of the CHOPPED Questionnaire: Outcomes per Question – Barriers
- Supplementary Fig. 4.1b — Outcomes of the CHOPPED Questionnaire: Outcomes per Question – Facilitators
- Supplementary Fig. 4.1c — Outcomes of the CHOPPED Questionnaire: Outcomes per question – Other
- Supplementary Fig. 4.2 — Outcomes of the CHOPPED Questionnaire: Outcomes per Factor

SUPPLEMENTARY TABLES

Supplementary Table 1 Laboratory and Clinical Parameters

Parameter	Median (IQR)		*N* (%)	
Heart rate (bpm)	80	(72–91)	337	(41)
Blood pressure (mmHg)				
Systolic	128	(115–145)	337	(41)
Diastolic	73	(64–82)	337	(41)
pO_2 (mmHg)	62	(48–76)	241	(30)
pCO_2 (mmHg)	39	(35–44)	241	(30)
Albumin (g/L)	40	(36–43)	518	(63)
Estimated glomerular filtration rate, CKD–EPI* (mL/min/1.73 m²)	90	(72–90)	801	(98)
Creatinine (µmol/L)	70	(58–84)	803	
HbA1c (mmol/mol)	42	(38–48)	169	(21)
Calcium (mmol/L)	2.4	(2.3–2.5)	556	(68)
Potassium (mmol/L)	4.2	(3.9–4.4)	764	(94)
Sodium (mmol/L)	138	(136–140)	766	(94)
Ferritin (µg/L)	224	(94–456)	263	(32)
Transferrin (µmol/L)	27.4	(21.4–32.1)	168	(21)
Hemoglobin (mmol/L)	8.2	(7.4–8.9)	795	(97)
Iron (µmol/L)	9	(5–15)	160	(20)

* The EHR system caps values at 90 mL/min/1.73 m², documenting all higher values as 90 mL/min/1.73 m².

Supplementary Table 2 Prescription Data and Identified PIMs

Class	No. of patients (*N*= 817) using ≥ 1 drug from class (%)		No. of patients (*N* = 817) with ≥ 1 PIM from class (%)	
**Allergy**				
Antihistamines – First generation	17	(2.1)	10	(58.8)
Antihistamines – Second generation	12	(1.5)	11	(91.7)
**Anti-inflammation**				
NSAIDs & Coxibs	123	(15.1)	123	(100.0)
Antigout drugs	23	(2.8)	3	(13.0)
Antimetabolites	8	(1.0)	0	(0.0)
mAb Immunosuppressants	1	(0.1)	0	(0.0)
**Antimicrobial**				
Antiprotozoals	4	(0.5)	3	(75.0)
Antibacterials	3	(0.4)	0	(0.0)
**Antithrombotics**				
Platelet aggregation inhibitors	225	(27.5)	225	(100.0)
Heparins	116	(14.2)	7	(6.0)
DOACs	87	(10.6)	87	(100.0)
Vitamin K antagonists	35	(4.3)	35	(100.0)
**Bone metabolism**				
Colecalciferol*	186	(22.8)	166	(89.2)
Bisphosphonates*	28	(3.4)	28	(100.0)
Denosumab	3	(0.4)	0	(0.0)
**CNS / Psychotropics**				
Benzodiazepines	211	(25.8)	211	(100.0)
Antidepressants	75	(9.2)	75	(100.0)
Antiepileptics	33	(4.0)	33	(100.0)
Antipsychotics	30	(3.7)	30	(100.0)
Methadone	16	(2.0)	16	(100.0)
Antivertigo	6	(0.7)	6	(100.0)
Antiparkinson	4	(0.5)	4	(100.0)
Antidementia	2	(0.2)	2	(100.0)
**Cardiovascular**				
RAAS-inhibitors	234	(28.6)	234	(100.0)
Beta blockers	227	(27.8)	227	(100.0)
Diuretics	127	(15.5)	127	(100.0)
Calcium channel blockers	121	(14.8)	121	(100.0)
Nitrates	79	(9.7)	48	(60.8)
Antiarrhythmics	20	(2.4)	19	(95.0)
Combinations	6	(0.7)	6	(100.0)
Others	3	(0.4)	2	(66.7)
**Corticosteroids (systemic)**				
Glucocorticosteroids	130	(15.9)	130	(100.0)
**Diabetes**				
Biguanides	88	(10.8)	84	(95.5)
Insulins	45	(5.5)	33	(73.3)
Sulfonylureas	30	(3.7)	28	(93.0)
SGLT2-inhibitors	4	(0.5)	3	(75.0)
DDP-4 inhibitors	3	(0.4)	3	(100.0)
GLP-1 analogues	3	(0.4)	2	(66.7)
Others	2	(0.2)	2	(100.0)
**Dyslipidemia**				
Statins	292	(35.7)	292	(100.0)
Ezetimibe	28	(3.4)	28	(100.0)
Ezetimibe/statin combination	8	(1.0)	8	(100.0)
Fibrates	4	(0.5)	4	(100.0)
PCSK9-inhibitors	4	(0.5)	4	(100.0)

Class	No. of patients (*N*= 817) using ≥ 1 drug from class (%)		No. of patients (*N* = 817) with ≥ 1 PIM from class (%)	
**Endocrine**				
Thyroid hormones	26	(3.2)	0	(0.0)
Estrogens and progestogens*	10	(1.2)	10	(0.0)
Androgens	3	(0.4)	3	(0.0)
Anti-estrogens	3	(0.4)	0	(0.0)
Anti-androgens	2	(0.2)	0	(0.0)
**Gastrointestinal**				
Proton pump inhibitors	390	(47.7)	328	(84.1)
Laxatives	290	(35.5)	87	(30.0)
Antiemetics	31	(3.8)	0	(0.0)
Antipropulsives	9	(1.1)	6	(66.7)
Antacids	7	(0.9)	7	(100.0)
H2-receptor antagonists	5	(0.6)	5	(100.0)
Mebeverine	5	(0.6)	0	(0.0)
Intestinal anti-inflammatories	4	(0.5)	0	(0.0)
**Pain management**				
Acetaminophen*	321	(39.3)	15	(4.7)
Strong opioids (short acting)	174	(21.3)	174	(100.0)
Strong opioids (long acting)	163	(20.0)	163	(100.0)
Weak opioids	46	(5.6)	46	(100.0)
GABA-analogues	26	(3.2)	26	(100.0)
Selective 5-HT1 agonists	3	(0.4)	1	(33.3)
Salicylic acid	2	(0.2)	2	(100.0)
**Respiratory**				
Selective β2-receptor agonists*	168	(20.6)	43	(25.6)
Anticholinergics*	97	(11.9)	97	(100.0)
Glucocorticosteroids*	88	(10.8)	52	(59.1)
Codeine	45	(5.5)	45	(100.0)
Adrenergics*	41	(5.0)	32	(78.0)
Mucolytics	10	(1.2)	10	(100.0)
Leukotriene receptor antagonist	1	(0.1)	0	(0.0)
PDE-4 inhibitors	1	(0.1)	0	(0.0)
**Urologicals**				
Alpha blockers	43	(5.3)	43	(100.0)
Antispasmodics	27	(3.3)	17	(63.0)
**Vitamins, Minerals and OTC drugs**				
Vitamin B (hydroxocobalamin, thiamine, -complex)	86	(10.5)	86	(100.0)
Folic acid	65	(8.0)	59	(90.8)
Iron supplements	34	(4.2)	34	(100.0)
Other minerals	21	(2.6)	18	(85.7)
Ascorbic acid	12	(1.5)	12	(100.0)
Multivitamins	5	(0.6)	3	(60.0)
Valerian	1	(0.1)	1	(100.0)

Table includes all medications in the OncoSTRIP checklist, organized by class. OTC = over-the-counter, PIMs = potentially inappropriate medication.

* Including combination products

Supplementary Table 3 Classification of Flagged PIMs

Class	No. of PIMs (% of total prescriptions)		No. of confirmed PIMs (% of total PIMs)		No. of unconfirmed PIMs (% of total PIMs)	
**Allergy**						
Antihistamines – First generation	11	(91.7)	11	(100.0)	0	(0.0)
Antihistamines – Second generation	10	(58.8)	10	(100.0)	0	(0.0)
**Anti-inflammation**						
NSAIDs & Coxibs	123	(100.0)	37	(30.1)	86	(70)
Antigout drugs	3	(1.5)	3	(100.0)	0	(0.0)
Antimetabolites	0	(0.0)	-	-	-	-
mAb Immunosuppressants	0	(0.0)	-	-	-	-
**Antimicrobial**						
Antiprotozoals	3	(75.0)	3	(100.0)	0	(0)
Antibacterials	0	(0.0)	-	-	-	-
**Antithrombotics**						
Platelet aggregation inhibitors	244	(100.0)	3	(1.2)	241	(98.8)
Heparins	7	(6.0)	0	(0)	7	(100.0)
DOACs	87	(100.0)	1	(1.1)	86	(98.9)
Vitamin K antagonists	35	(100.0)	0	(0)	35	(100.0)
**Bone metabolism**						
Colecalciferol*	173	(89.2)	0	(0)	173	(100.0)
Bisphosphonates*	28	(100.0)	0	(0)	28	(100.0)
Denosumab	0	(0)	-	-	-	-
**CNS / Psychotropics**						
Benzodiazepines	261	(100.0)	205	(78.5)	56	(21.5)
Antidepressants	86	(100.0)	69	(80.2)	17	(19.8)
Antiepileptics	40	(100.0)	8	(20.0)	32	(80.0)
Antipsychotics	36	(100.0)	29	(80.6)	7	(19.4)
Methadone	17	(100.0)	8	(47.1)	9	(52.9)
Antivertigo	7	(100.0)	2	(28.6)	5	(71.4)
Antiparkinson	5	(100.0)	4	(80.0)	1	(20.0)
Antidementia	2	(100.0)	2	(100.0)	0	(0.0)
**Cardiovascular**						
RAAS-inhibitors	243	(100.0)	1	(0.4)	242	(99.6)
Beta blockers	236	(100.0)	6	(2.5)	230	(97.5)
Diuretics	147	(100.0)	9	(6.1)	138	(93.9)
Calcium channel blockers	121	(100.0)	4	(3.3)	117	(96.7)
Nitrates	57	(55.3)	51	(89.5)	6	(10.5)
Antiarrhythmics	20	(95.2)	8	(40.0)	12	(60.0)
Combinations	6	(100.0)	3	(50.0)	3	(50.0)
Others	2	(66.7)	0	(0.0)	2	(100.0)
**Corticosteroids (systemic)**						
Glucocorticosteroids	131	(100.0)	80	(61.1)	51	(38.9)
**Diabetes**						
Biguanides	87	(95.6)	53	(60.9)	34	(39.1)
Insulins	48	(67.6)	12	(25.0)	36	(75.0)
Sulfonylureas	30	(93.8)	6	(20.0)	24	(80.0)
SGLT2-inhibitors	3	(75.0)	0	(0.0)	3	(100.0)
DDP-4 inhibitors	3	(100.0)	0	(0.0)	3	(100.0)
GLP-1 analogues	2	(66.7)	0	(0.0)	2	(100.0)
Others	2	(100.0)	0	(0.0)	2	(100.0)
**Dyslipidemia**						
Statins	294	(100.0)	294	(100.0)	0	(0.0)
Ezetimibe	28	(100.0)	28	(100.0)	0	(0.0)
Ezetimibe/statin combination	8	(100.0)	8	(100.0)	0	(0.0)

Class	No. of PIMs (% of total prescriptions)		No. of confirmed PIMs (% of total PIMs)		No. of unconfirmed PIMs (% of total PIMs)	
Fibrates	4	(100.0)	4	(100.0)	0	(0.0)
PCSK9-inhibitors	4	(100.0)	4	(100.0)	0	(0.0)
**Endocrine**						
Thyroid hormones	0	(0.0)	-	-	-	-
Estrogens and progestogens*	10	(90.9)	0	(0.0)	10	(100.0)
Androgens	3	(100.0)	0	(0.0)	3	(100.0)
Anti-estrogens	0	(0.0)	-	-	-	-
Anti-androgens	0	(0.0)	-	-	-	-
**Gastrointestinal**						
Proton pump inhibitors	328	(84.1)	230	(70.1)	98	(29.9)
Laxatives	96	(31.3)	4	(4.2)	92	(95.8)
Antiemetics	0	(0.0)	-	-	-	-
Antipropulsives	6	(66.7)	6	(100.0)	0	(0.0)
Antacids	7	(100.0)	0	(0.0)	7	(100.0)
H2-receptor antagonists	5	(100.0)	4	(80.0)	1	(20.0)
Mebeverine	0	(0.0)	-	-	-	-
Intestinal anti-inflammatories	0	(0.0)	-	-	-	-
**Pain management**						
Acetaminophen*	15	(4.6)	15	(100.0)	0	(0.0)
Strong opioids (short acting)	177	(100.0)	133	(75.1)	44	(24.9)
Strong opioids (long acting)	173	(100.0)	138	(79.8)	35	(20.2)
Weak opioids	48	(100.0)	42	(87.5)	6	(12.5)
GABA-analogues	27	(100.0)	0	(0.0)	27	(100.0)
Selective 5-HT1 agonists	1	(33.3)	1	(100.0)	0	(0.0)
Salicylic acid	2	(100.0)	0	(0.0)	2	(100.0)
**Respiratory**						
Selective β2-receptor agonists*	43	(23.2)	29	(67.4)	14	(57.6)
Anticholinergics*	103	(100.0)	72	(69.9)	31	(30.1)
Glucocorticosteroids*	54	(59.3)	7	(13.0)	47	(87.0)
Codeine	45	(100.0)	2	(4.4)	43	(95.6)
Adrenergics*	33	(76.7)	14	(42.4)	19	(57.6)
Mucolytics	10	(100.0)	10	(100.0)	0	(0.0)
Leukotriene receptor antagonist	0	(0.0)	-	-	-	-
PDE-4 inhibitors	0	(0.0)	-	-	-	-
**Urologicals**						
Alpha blockers	43	(100.0)	1	(2.3)	42	(97.7)
Antispasmodics	17	(60.7)	9	(52.9)	8	(47.1)
**Vitamins, Minerals and OTC drugs**						
Vitamin B (hydroxocobalamin, thiamine, -complex)	106	(100.0)	0	(0.0)	106	(100.0)
Folic acid	59	(89.4)	0	(0.0)	59	(100.0)
Iron supplements	34	(100.0)	1	(2.9)	33	(97.1)
Other minerals	19	(86.4)	6	(31.6)	13	(68.4)
Ascorbic acid	12	(100.0)	0	(0.0)	12	(100.0)
Multivitamins	3	(60.0)	0	(0.0)	3	(60.0)
Valerian	1	(100.0)	0	(0.0)	1	(100.0)
**Total**	**4,134**		**1,686**		**2,448**	

Table includes all medications in the OncoSTRIP checklist, organized by class. OTC = over-the-counter, PIMs = potentially inappropriate medication.

* Including combination products

Supplementary Table 4 Data Quality and Reliability

Participant Group	Sample size (*n*)	Cronbach's Alpha (α) [95%CI]	ICC* [95%CI]	ICC, F-test (df, *p*)	Bootstrap, mean score [95%CI]
Patients	37	0.83 [0.75–0.90]	0.064 [0.03–0.12]	F(36,149) = 3.69 , *p* = < .001	3.02 [2.89–3.14]
Caregivers	15	0.84 [0.69–0.94]	0.020 [-0.01–0.10]	F(14,253) = 1.48 , *p* = .12	3.12 [3.00–3.24]
Clinicians	24	0.81 [0.68–0.90]	0.049 [0.02–0.11]	F(23,344) = 3.88 , *p* = < .001	3.34 [3.24–3.47]
Pharmacists	24	0.85 [0.41–0.92]	0.045 [0.02–0.10]	F(23,276) = 4.1 , *p* = < .001	3.19 [3.09–3.31]

* Using Single-Rating, Absolute-Agreement, 2-Way Random-Effects Model

SUPPLEMENTARY FIGURES

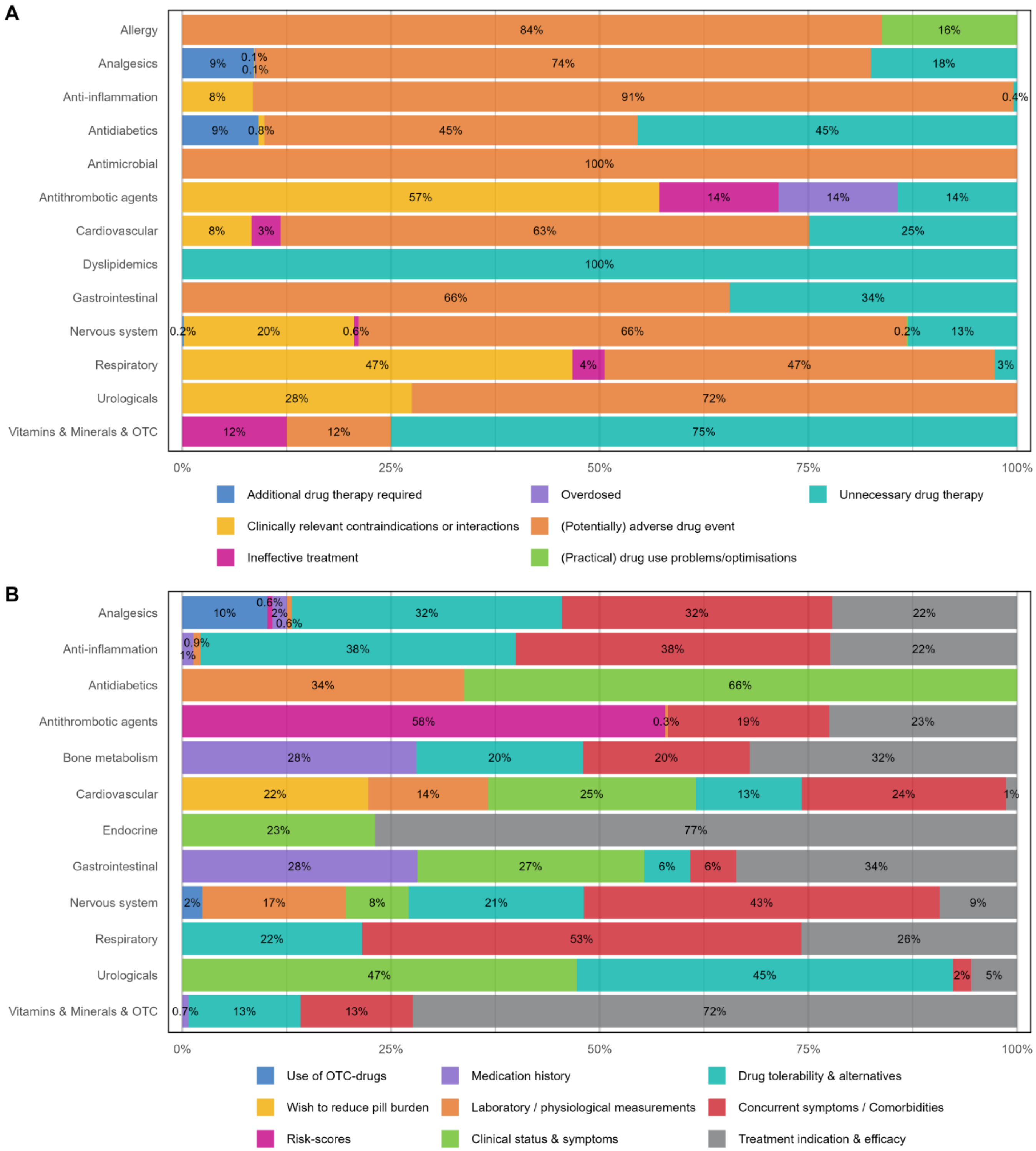

Supplementary Fig. 1. Clinical Rationale and Data Limitations for Identified PIMs

Analysis of pharmacotherapeutic categories flagged by the OncoSTRIP algorithm, stratified by the clinical motivation for "confirmed" PIM status (**a**) and the specific missing clinical context that precluded definitive determination for "unconfirmed" PIM status (**b**).

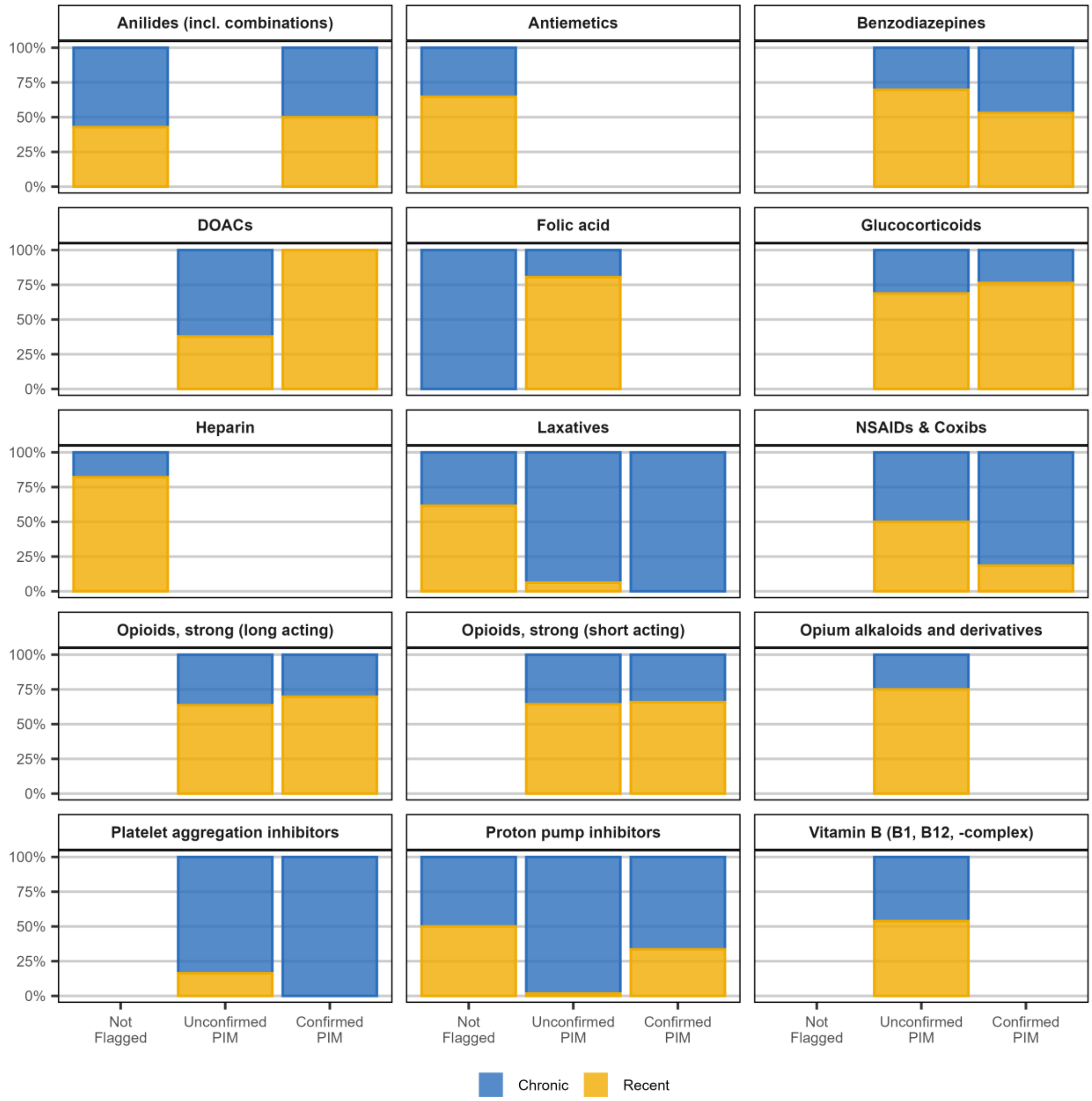

Supplementary Fig. 2 Temporal Initiation Patterns of Medications Flagged by the OncoSTRIP Algorithm. Proportions of chronic versus recent initiation for the most frequent drug groups identified by the computerized OncoSTRIP tool, stratified by screening status as “Confirmed PIM”, “Unconfirmed PIM” or “Not Flagged”.

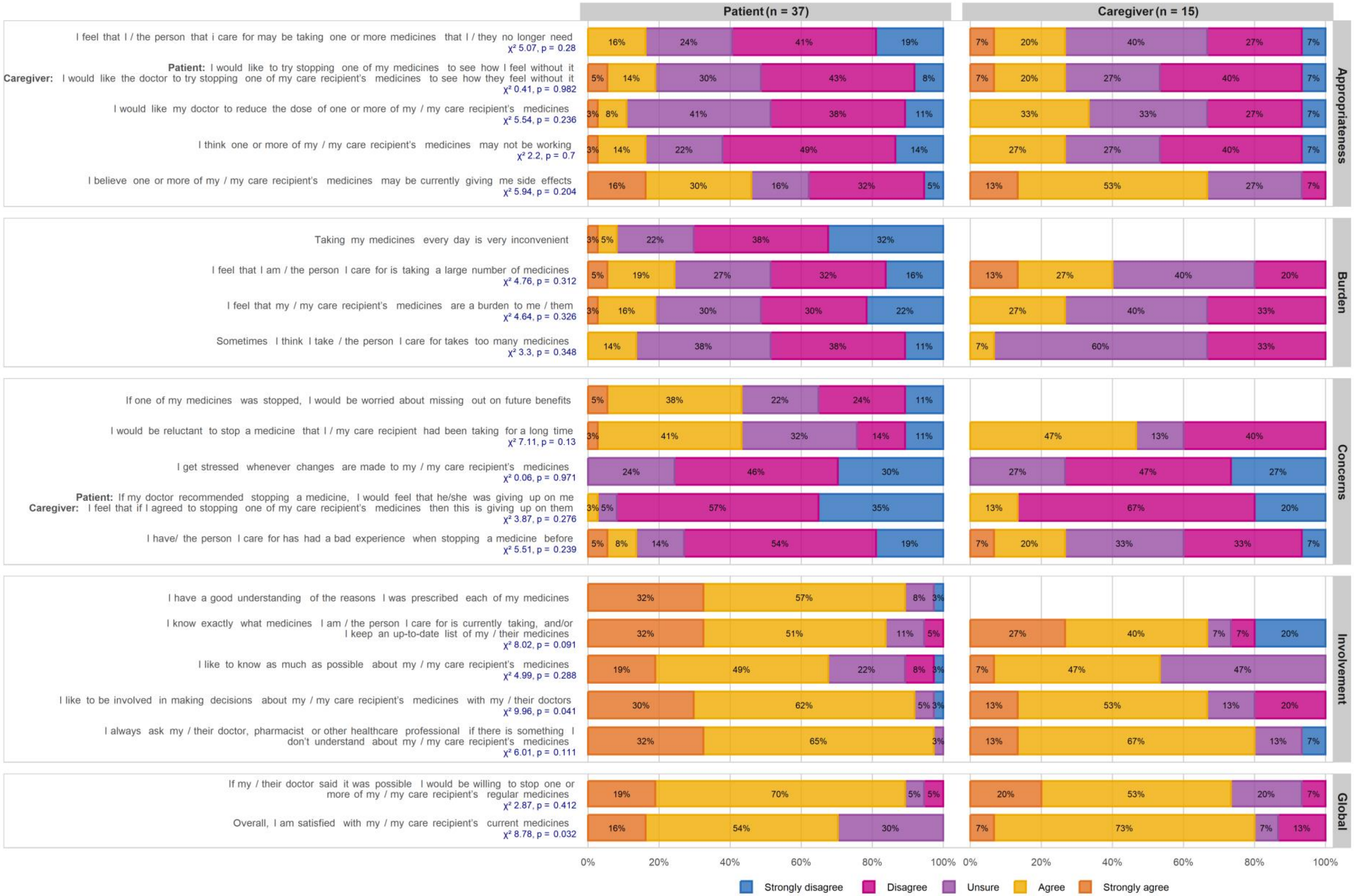

Supplementary Fig. 3.1 Outcomes of the rPATD Questionnaire: Outcomes per Question. Differences between respondent groups were evaluated using the Chi-square test.

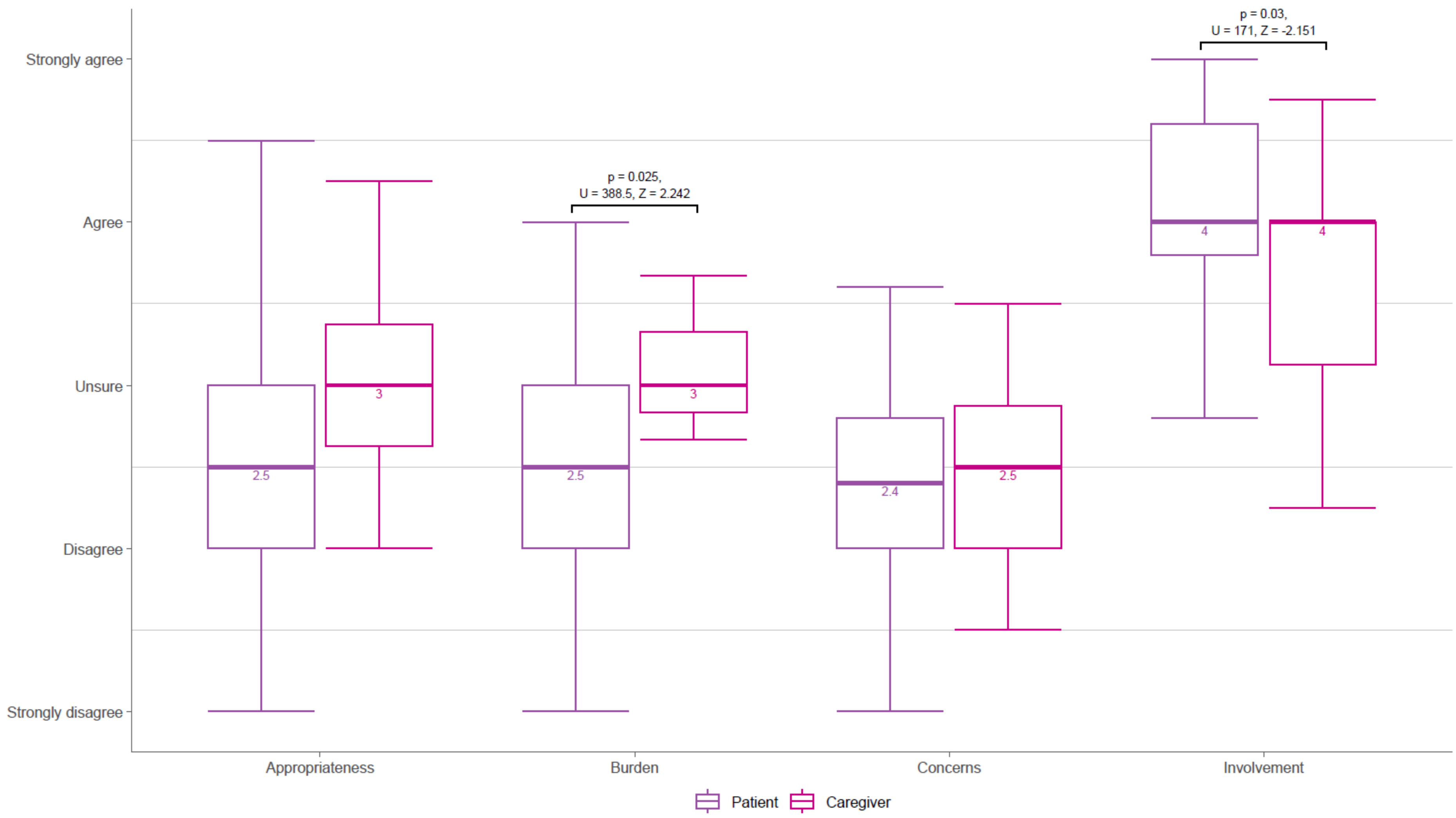

Supplementary Fig. 3.2 Outcomes of the rPATD Questionnaire: Outcomes per Factor. Differences between respondent groups were evaluated using the Mann-Whitney U test.

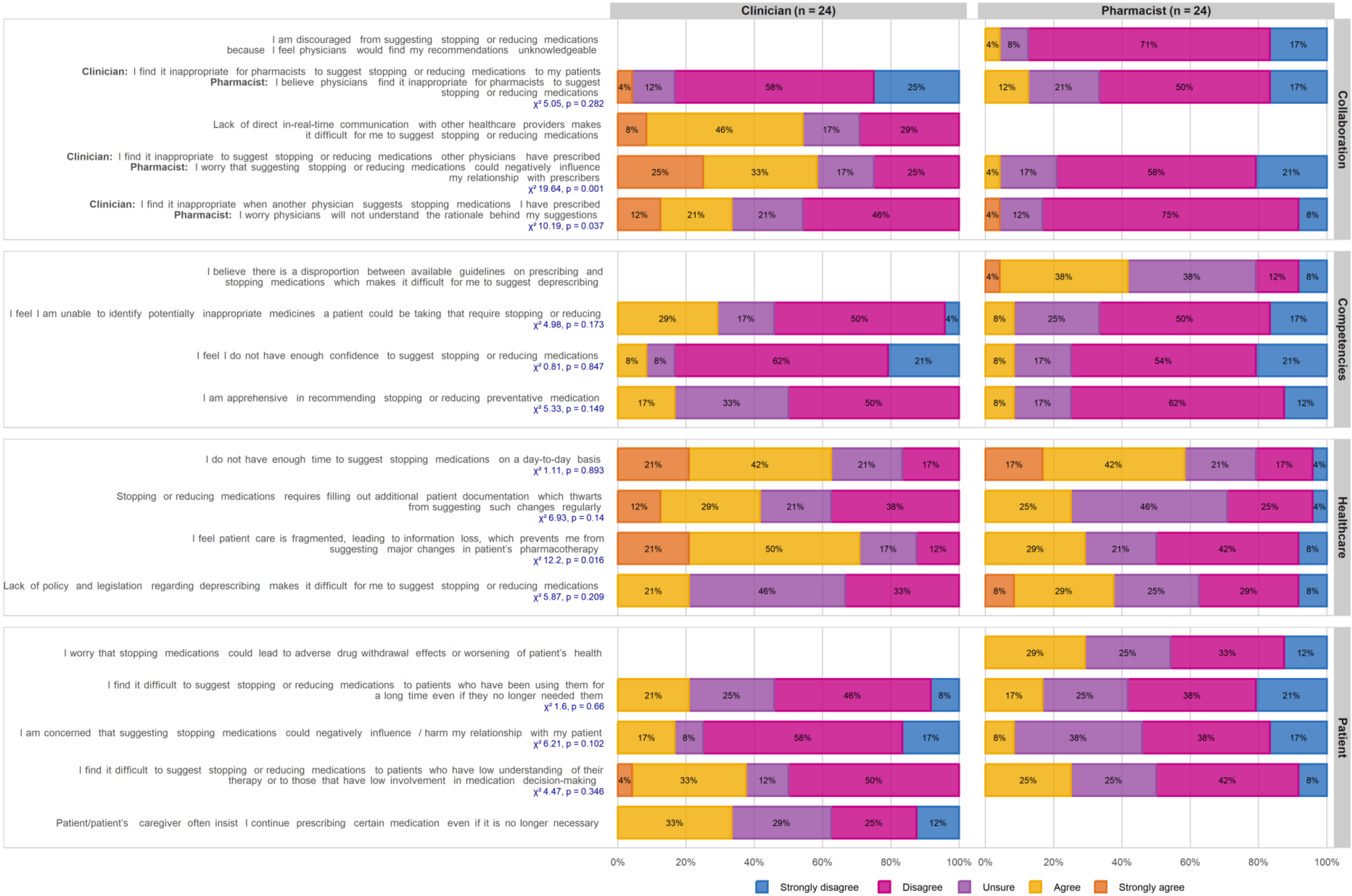

Supplementary Fig. 4.1a Outcomes of the CHOPPED Questionnaire: Outcomes per Question – Barriers. Differences between respondent groups were evaluated using the Chi-square test.

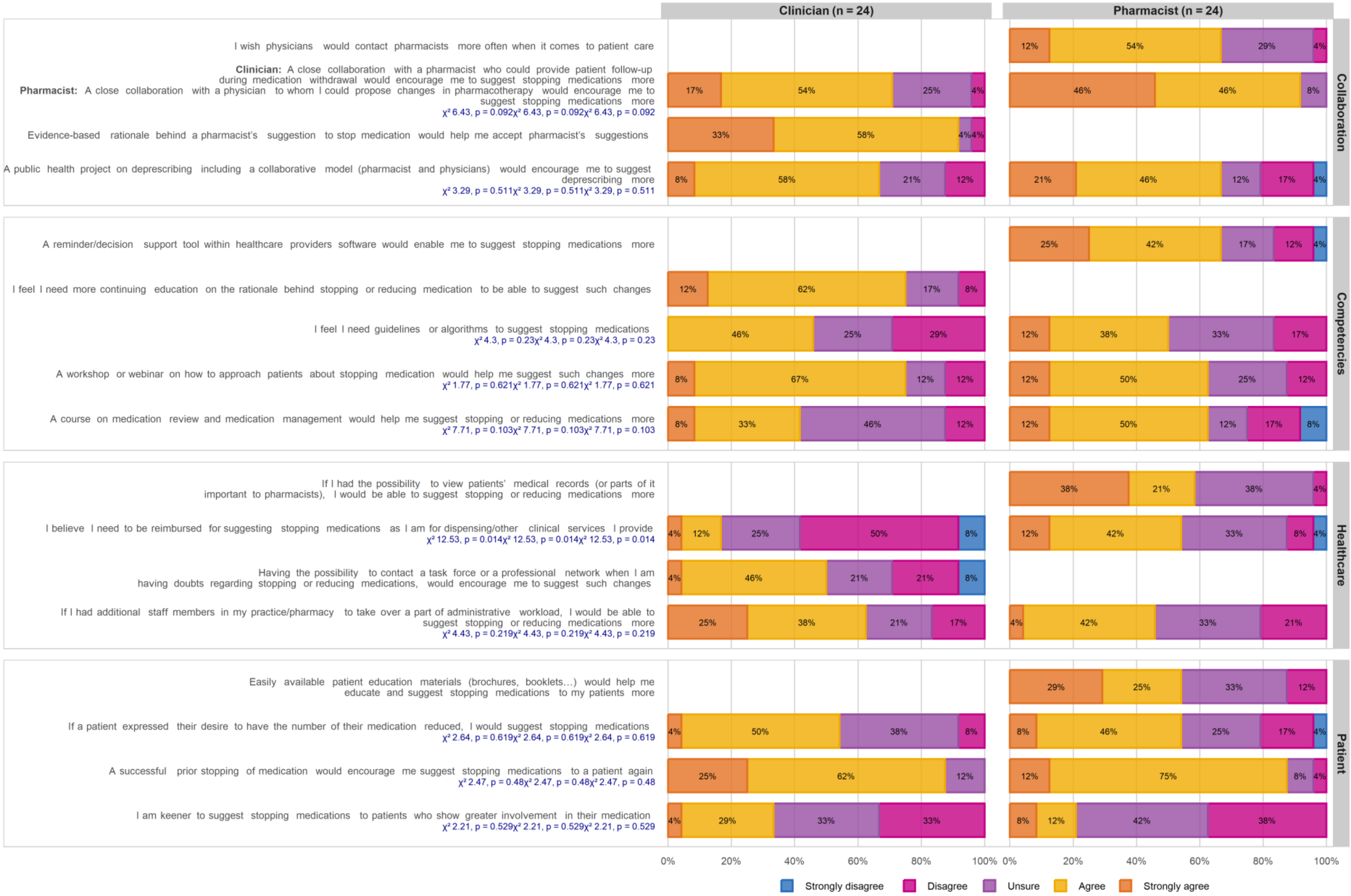

Supplementary Fig. 4.1b Outcomes of the CHOPPED Questionnaire: Outcomes per Question – Facilitators. Differences between respondent groups were evaluated using the Chi-square test.

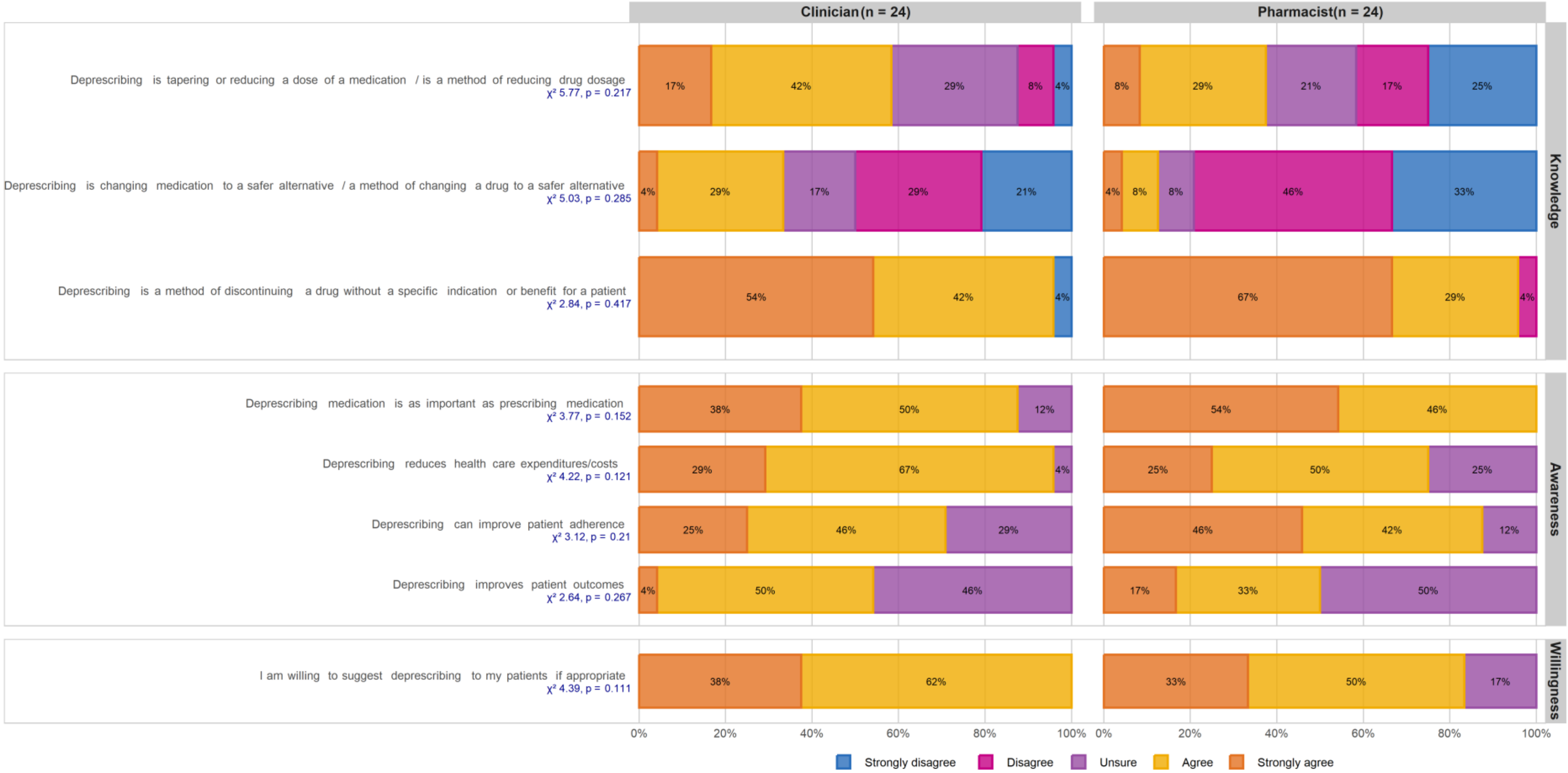

Supplementary Fig. 4.1c Outcomes of the CHOPPED Questionnaire: Outcomes per Question – Other. Differences between respondent groups were evaluated using the Chi-square test.

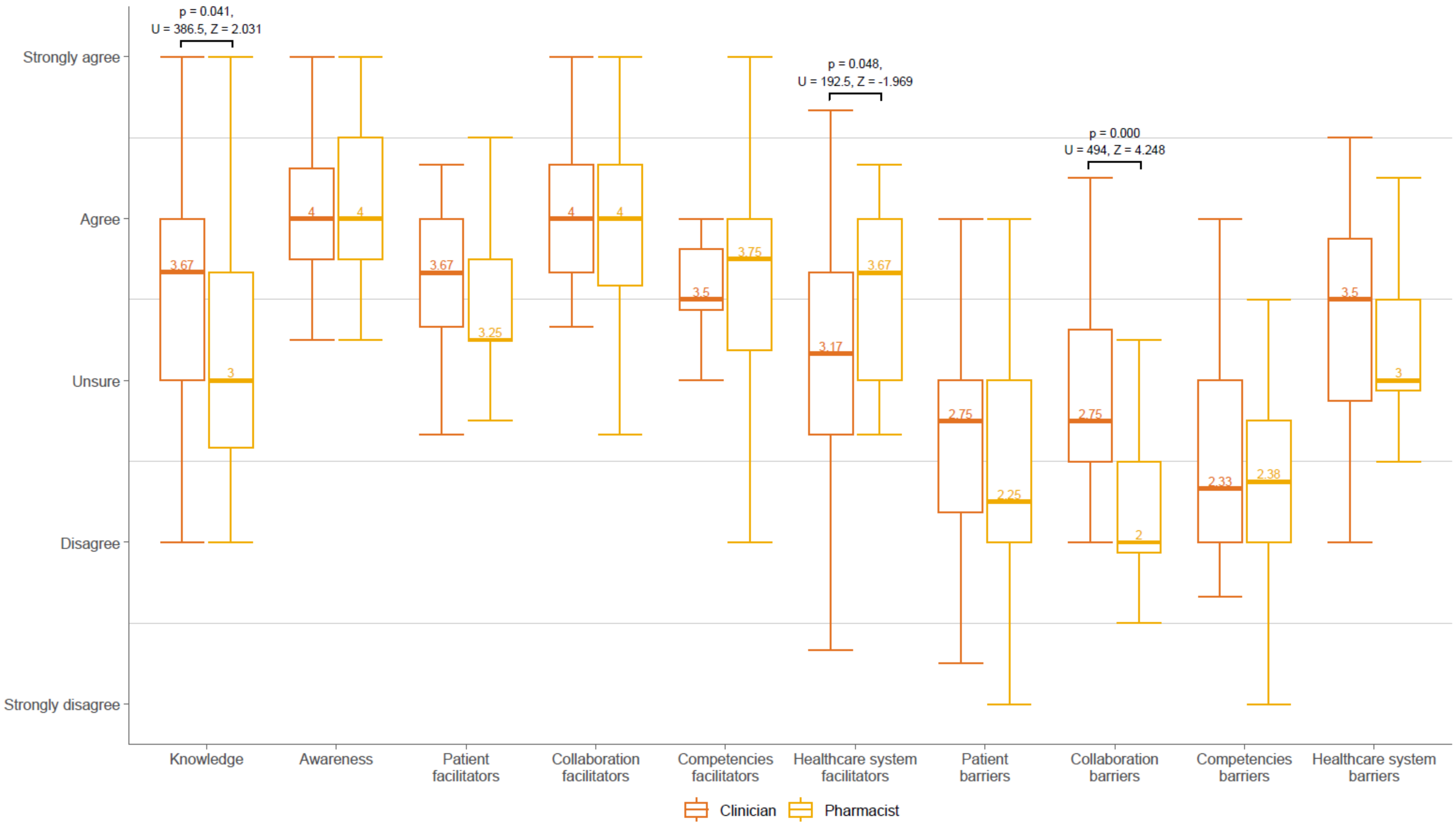

Supplementary Fig. 4.2 Outcomes of the CHOPPED Questionnaire: Outcomes per Factor. Differences between respondent groups were evaluated using the Mann-Whitney U test.
